# A child with distal (type 1) renal tubular acidosis presenting with progressive gross motor developmental regression and acute paralysis

**DOI:** 10.1186/s13104-017-2949-2

**Published:** 2017-11-25

**Authors:** Randula Ranawaka, Kavinda Dayasiri, Manoji Gamage

**Affiliations:** 1 Department of Paediatrics, Faculty of Medicine, University of Colombo, Kynsey Road, Colombo 8, Sri Lanka; 2 Professorial Paediatric Unit, Lady Ridgeway Hospital for Children, Colombo 08, Sri Lanka; 3 Nutrition Unit, Lady Ridgeway Hospital for Children, Colombo, Sri Lanka

**Keywords:** Distal (type 1) renal tubular acidosis, Hypokalemia, Acute flaccid paralysis, Proximal myopathy

## Abstract

**Background:**

Distal (Type 1) renal tubular acidosis (dRTA) is characterized by inability to secrete hydrogen irons from the distal tubule. The aetiology of dRTA is diverse and can be either inherited or acquired. Common clinical presentations of dRTA in the paediatric age group include polyuria, nocturia, failure to thrive, constipation, abnormal breathing and nephrolithiasis. Though persistent hypokalemia is frequently seen in dRTA, hypokalemic muscular paralysis is uncommon and rarely described in children.

**Case presentation:**

Three and a half years old girl was referred for evaluation of progressive loss of gross motor milestones over 6 months and acute episode of paralysis. Her other developmental domains were age appropriate. Notably, there was no history of polyuria, polydipsia, nocturia and abnormal breathing. Physical examination revealed proximal myopathy (waddling gait and positive Gower’s sign), diminished lower limb reflexes and muscle tone. Her serum potassium was low (2.1 meq/l) and she was subsequently investigated for hypokalemic paralysis. Diagnosis of distal renal tubular acidosis was made, based on hypokalemic hyperchloremic metabolic acidosis with normal anion gap, high urine pH, borderline hypercalciuria, medullary nephrocalcinosis and exclusion of other differential diagnosis. The child showed complete symptomatic recovery upon commencement of standard treatment for distal renal tubular acidosis.

**Conclusions:**

This case report highlights the importance of considering hypokalemia and renal tubular acidosis in the differential diagnosis of acute flaccid paralysis and proximal myopathy. Early diagnosis will prevent costly investigations and enable rapid clinical recovery in the affected child.

## Background

Distal (Type 1) renal tubular acidosis (dRTA) is characterized by inability to secrete hydrogen irons from the distal tubule. It was first described in 1946 [1] and characterized by a clinical syndrome consisting of hypokalemic, hyperchloremic metabolic acidosis with normal anion gap, high urine pH (> 5.5), nephrocalcinosis and nephrolithiasis [2]. The aetiology of dRTA is diverse and can be either inherited or acquired. The genetic basis of dRTA was described recently as a defect in urinary excretion of ammonium [3].

Common clinical presentations of dRTA in the paediatric age group include polyuria, failure to thrive, constipation, abnormal breathing and nephrolithiasis. Though persistent hypokalemia is frequently seen in dRTA, hypokalemic muscular paralysis is uncommon and rarely described in children [4]. This case report has described a child with distal (type 1) renal tubular acidosis who presented with gross motor developmental regression and acute episode of paralysis.

## Case presentation

Three and a half years old girl was referred for evaluation of progressive loss of gross motor milestones over 6 months and acute episode of paralysis. She was first born to healthy non-consanguineous parents and without family history of motor developmental disorders. Parents were concerned by progressively worsening of difficulty in walking, climbing and standing from sitting position. Her other developmental domains were age appropriate. Notably, there was no history of polyuria, polydipsia and rapid breathing. Physical examination revealed proximal myopathy (waddling gait and positive Gower’s sign), diminished knee reflex and reduced muscle tone.

Initial investigations revealed normal serum creatine phosphokinase (89 U/l), TSH (thyroid stimulating hormone) (3.73 µIU/ml), electromyogram and nerve conduction studies. She also had normal serum calcium (2.3 mmol/l), magnesium (0.8 mmol/l), lactate (0.57 mmol/l) and ammonia (47 µg/dl). Muscle biopsy was suggestive of non-specific myopathy and did not reveal red ragged fibers, mitochondrial disruption and necrosis. MRI brain and spine showed normal findings. Her serum potassium was low (2.1 meq/l) and she was subsequently investigated for hypokalemic paralysis.

Venous blood gases showed metabolic acidosis (pH − 7.3, HCO^3−^ 19 meq/l) with a normal anion gap (8 meq/l). Urine pH was 6.5. Urine was negative for reducing substances and protein. Renal ultrasound revealed bilateral nephrocalcinosis. Serum creatinine (32 µmol/l) was normal. She had hyperchloremia (serum chloride—113 meq/l). Urine calcium/creatinine ratio was high (0.72 mmol/mmol). Tubular resorption of phosphate was normal (> 80%).

Diagnosis of distal renal tubular acidosis was made, based on hypokalemic hyperchloremic metabolic acidosis with normal anion gap, high urine pH, hypercalciuria, medullary nephrocalcinosis and exclusion of other differential diagnosis. The child showed complete symptomatic recovery and achieved all age appropriate gross motor milestones upon commencement of standard treatment, poly-citra for distal renal tubular acidosis.

## Discussion and conclusions

Distal renal tubular acidosis in children is mostly hereditary [5]. It can however, occur secondary to obstructive uropathies, drugs and toxin exposure, and autoimmune diseases [6]. Potassium depletion in dRTA can be secondary to number of genetic defect in cellular metabolism manifested as autosomal dominant or recessive [7] condition.

dRTA in the paediatric age group commonly presents with polyuria, constipation, failure to thrive and nephrolithiasis. Hypokalemic paralysis and progressive weakness are rare manifestations reported in children [8] and data on incidence are not available in literature [5]. Hypokalemic paralysis had been periodic in several reported cases of dRTA [9]. Progressive proximal myopathy as the presentation of hypokalemia in dRTA is very unusual and had been previously reported in association with polymyositis [10]. The unusual presentation was misinterpreted in the initial stage as a primary neuromuscular disorder and the diagnosis was revealed only after extended evaluation. Limited literature has indicated that this atypical presentation can always be misinterpreted as a neurological disorder highlighting the importance of the need of initial evaluation with serum electrolytes [4].

Levels of calcium and phosphate are usually normal in dRTA. However, rickets is common in untreated dRTA because of bone resorption to buffer chronic metabolic acidosis [11]. Mortality following hypokalemic paralysis is secondary to respiratory failure and cardiac arrhythmias [8]. Management of dRTA includes alkali and potassium replacement along with the treatment of the underlying disorder.

This case report highlights the importance of considering hypokalemia and renal tubular acidosis in the differential diagnosis of acute flaccid paralysis and proximal myopathy. Early diagnosis will prevent costly investigations and enable rapid clinical recovery in the affected child.

## Abbreviation

dRTA: distal (type 1) renal tubular acidosis
